# Supplementary material for: Interleukin-36 upregulates type-I interferon responses in systemic lupus erythematosus by promoting the accumulation of self-nucleic acids
Source: Front Immunol. 2026 Jan 13;16:1727524. doi: 10.3389/fimmu.2025.1727524 (PMC12835401; doi:10.3389/fimmu.2025.1727524)
Supplement: Supplementary file 1 [file DataSheet1.docx]

Supplementary Material

1. Supplementary Figures


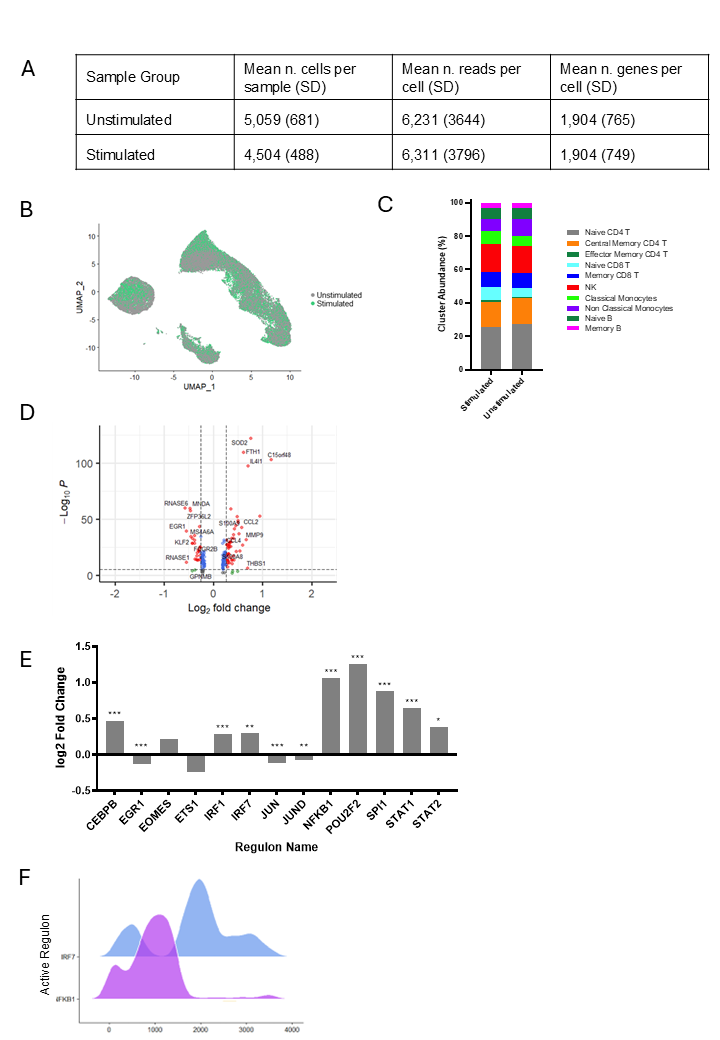


**Supplementary Figure S1:** Single-cell RNA sequencing of IL-36 stimulated PBMCs (A) Single-cell RNA sequencing output. SD, standard deviation. (B) UMAP of 51,691 single cells showing that clustering is not affected by treatment status. (C) Volcano plot showing the DEG detected in non-classical monocytes following IL-36 stimulation. Horizontal and vertical dashed lines show the thresholds for statistical significance (FDR<0.05) and fold change (log2foldchange>log1.2), respectively. Differentially expressed genes meeting both thresholds are represented by red dots, those that only meet the significance threshold are shown as blue dots. (D) Bar plots summarizing the changes in activity for the 13 regulons detected in non-classical monocytes. Positive log2foldchanges indicate increased activity in samples treated with IL-36. **P*<0.05; **P<0.01; ****P*<0.001 (χ^2^ test followed by Bonferroni correction). (E) Slingshot pseudotime analysis of classical monocytes displaying NFKB1 or IRF7 regulon activation.


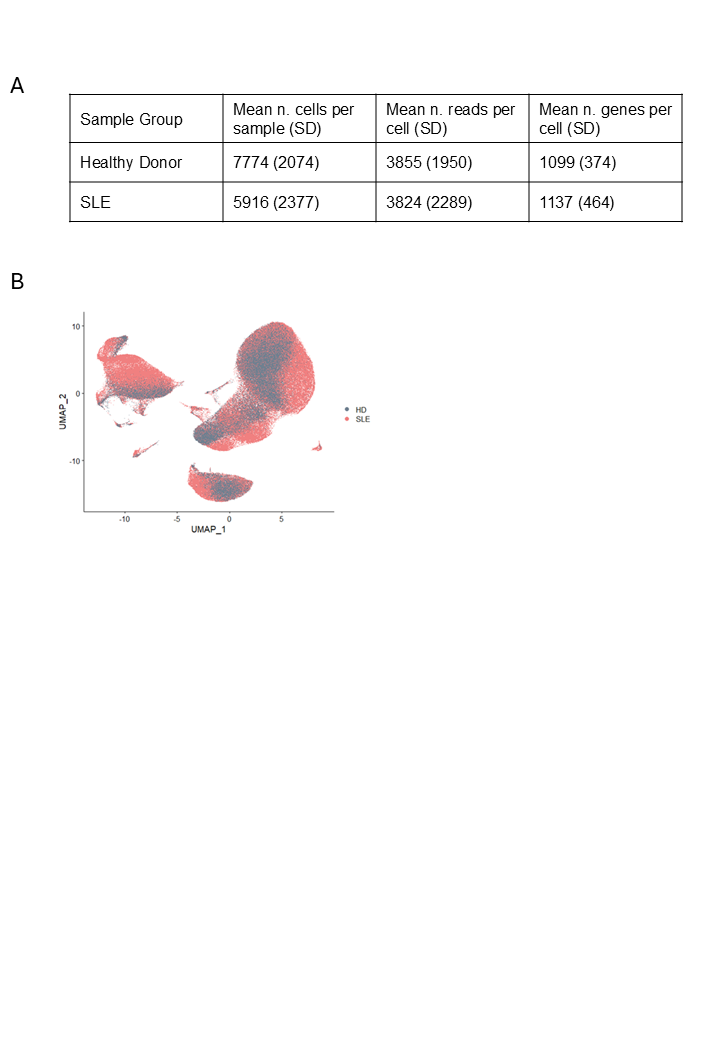


C


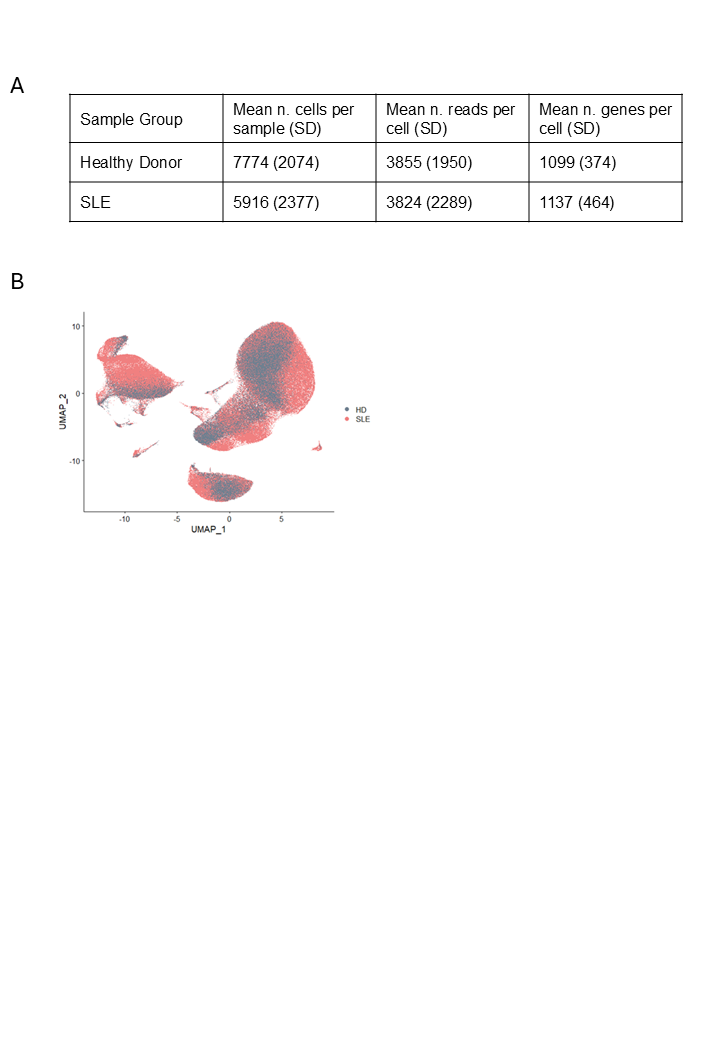

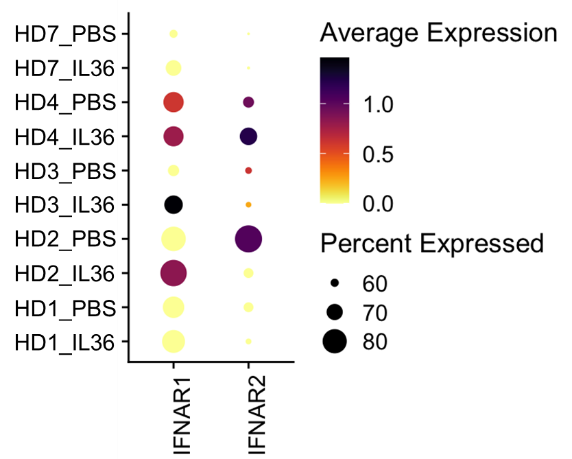


**Supplementary Figure S2:** Analysis of single-cell RNA sequencing data generated in SLE cases and healthy donors (A) Single-cell RNA sequencing output. SD, standard deviation. (B) UMAP of 280,725 single cells showing that clustering is not influenced by participant group. (C) Plot showing that the expression of IFNAR genes in NK cells is mostly unaffected by IL-36 treatment. HD, healthy donor.


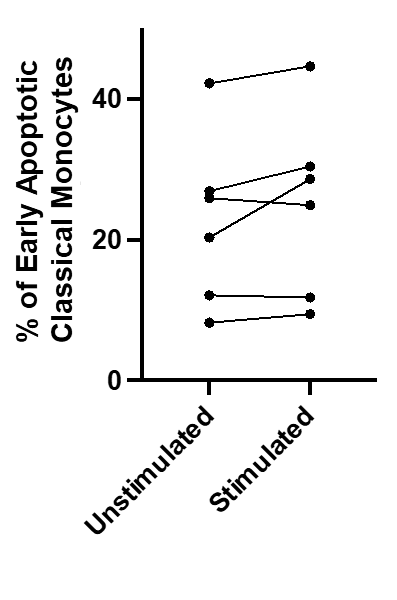


**Supplementary Figure S3**: Effects of IL-36 treatment on monocyte apoptosis. The paired dot plot shows that IL-36 treatment has a modest effect on the proportion of classical monocytes identified as early apoptotic cells (Annexin V+/PI- population) (n=6 donors). Each line represents one donor. The representative flow-cytometry plot is shown in Figure 3C.

1. **Supplementary Tables**

**Supplementary Table S1:** Genes underlying the IL-36 score

| **Gene Name** | **avg_log2FC** | **FDR** |
| --- | --- | --- |
| *C15orf48* | 2.01 | 6.2x10^-195^ |
| *SOD2* | 1.62 | 9.2x10^-197^ |
| *THBS1* | 1.50 | 7.6x10^-93^ |
| *CCL2* | 1.29 | 3.5x10^-51^ |
| *CCL4* | 1.14 | 1.3x10^-53^ |
| *ADA* | 1.13 | 3.0x10^-94^ |
| *CCL3* | 1.07 | 3.6x10^-39^ |
| *IL1B* | 1.05 | 3.9x10^-20^ |
| *SLC39A8* | 0.99 | 6.5x10^-83^ |

FC; fold change, FDR; false discovery rate.

**Supplementary Table S2:** Real-time PCR primers

| **Target gene** | **Primer sequence (5’ to 3’)** |
| --- | --- |
| *GAPDH* | F: CGGAGTCAACGGATTTGGTC  R: AATGAAGGGGTCATTGATGGCA |
| *RNASE1* | F: AGCTGCAGATCCAGGCTTT  R: CTGCCGCTGGAATTTCTTGG |
| *RNASE6* | F: GAGACCAGAAAAGATGGTGCT  R: GAGCCTTGGTGAGACGCTTA |
| *RNASET2* | F: ATACATGGACTATGGCCCGA  R: TGCGATTGGGAAACGAGTGA |

F, forward; R, reverse.

**Supplementary Table S3:** Flow Cytometry Antibodies

| **Fluorochrome** | **Target** | **Clone** | **Cat. No** | **Supplier** | **Dilution** |
| --- | --- | --- | --- | --- | --- |
| AF700 | CD14 | 63D3 | 367114 | BioLegend | 1:100 |
| APC | CD16 | 3G8 | 302011 | BioLegend | 1:50 |
| FITC | Annexin V | N/A | 33-1200 | Thermo Fisher | N/A |

AF700, Alexa Fluor 700; APC, Allophycocyanin; FITC, Fluorescein isothiocyanate; N/A, not applicable.

**Supplementary Table S4**: SLE dataset clinical characteristics

| **Total number of cases** | n=33 |
| --- | --- |
| **SLEDAI** |  |
| Score, mean (range) | 4.9 (0-19) |
| SLEDAI 0-1, n (%) | 8 (24.2%) |
| SLEDAI ≥2, n (%) | 25 (75.8%) |
| **SLEDAI components,** n (%) |  |
| Anti-dsDNA antibodies | 23 (69.7%) |
| Skin involvement and/or arthritis | 7 (21.2%) |
| Renal involvement | 8 (24.2%) |
| **Medications**, n (%) |  |
| None/HCQ alone | 9 (24.2%) |
| GC +/- HCQ | 8 (60.6%) |
| MTX/MMF +/-HCQ +/-GC | 16 (48.5%) |

GC, glucocorticoids; HCQ, hydroxychloroquine; MMF, mycophenolate; MTX, methotrexate; SLEDAI, systemic lupus erythematosus disease activity index. Adapted from (1)

1. **Supplementary reference**

1. Nehar-Belaid D, Hong S, Marches R, Chen G, Bolisetty M, Baisch J, et al. Mapping systemic lupus erythematosus heterogeneity at the single-cell level. Nat Immunol. 2020;21(9):1094-106.
